# Benchmark Ab Initio Characterization of the Abstraction and Substitution Pathways of the Cl + CH_3_CN Reaction

**DOI:** 10.1021/acs.jpca.2c01376

**Published:** 2022-04-28

**Authors:** Petra Tóth, Tímea Szűcs, Gábor Czakó

**Affiliations:** MTA-SZTE Lendület Computational Reaction Dynamics Research Group, Interdisciplinary Excellence Centre and Department of Physical Chemistry and Materials Science, Institute of Chemistry, University of Szeged, Rerrich Béla tér 1, Szeged H-6720, Hungary

## Abstract

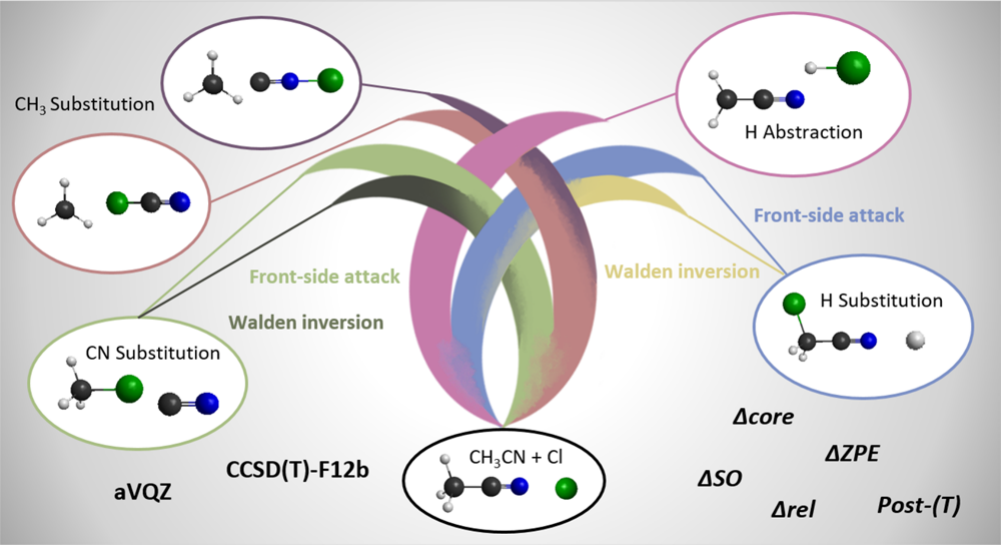

We investigate the reaction pathways of the Cl + CH_3_CN system: hydrogen abstraction, methyl substitution, hydrogen substitution, and cyanide substitution, leading to HCl + CH_2_CN, ClCN/CNCl + CH_3_, ClCH_2_CN + H, and CH_3_Cl + CN, respectively. Hydrogen abstraction is exothermic and has a low barrier, whereas the other channels are endothermic with high barriers. The latter two can proceed via a Walden inversion or front-side attack mechanism, and the front-side attack barriers are always higher. The C-side methyl substitution has a lower barrier and also a lower endothermicity than the N-side reaction. The computations utilize an accurate composite ab initio approach and the explicitly correlated CCSD(T)-F12b method. The benchmark classical and vibrationally adiabatic energies of the stationary points are determined with the most accurate CCSD(T)-F12b/aug-cc-pVQZ energies adding further contributions of the post-(T) and core correlation, scalar relativistic effects, spin–orbit coupling, and zero-point energy corrections. These contributions are found to be non-negligible to reach subchemical accuracy.

## 1 Introduction

One of the main goals of chemistry is to understand the mechanisms of reactions at the deepest atomic and molecular levels. For small, few-atom systems, such as the reactions of the Cl atom with H_2_, H_2_O, NH_3_, and CH_4_ molecules, modern quantum chemical methods are capable of determining the stationary-point energetics with subchemical accuracy, thereby revealing possible reaction pathways and guiding various experiments, potential energy surface (PES) developments, and reaction dynamics studies.^1−20^ In addition to the above-mentioned fundamental benchmark systems, reactions of more complex molecules such as C_2_H_6_,^21−25^ CH_3_OH,^26−29^ and CH_3_NH_2_^30−32^ have also attracted significant attention. The main reaction pathway of these processes is hydrogen abstraction forming a HCl molecule and a radical product. However, recent studies^23,24,32^ revealed that several other product channels are also possible such as H + CH_2_ClNH_2_, H + CH_3_NHCl, NH_2_ + CH_3_Cl, and CH_3_ + NH_2_Cl in the case of the Cl + CH_3_NH_2_ reaction.^32^ Quantum chemical computations can determine the reaction enthalpies and barrier heights for these processes, thereby revealing their thermodynamic and kinetic requirements, respectively. Even if some of these pathways may proceed over high barriers and cannot occur at standard thermal conditions, they may be accessible by crossed-beam techniques at hyperthermal collision energies as Minton and co-workers^33^ did in the case of the O(^3^P) + CH_4_/C_2_H_6_/C_3_H_8_ reactions revealing novel reaction pathways such as H substitution, CH_3_ substitution, etc.

In the present electronic structure study, we focus on the Cl + CH_3_CN reaction, which has also attracted considerable experimental and theoretical attention in the past few decades.^34−42^ Rate constants for the Cl + CH_3_CN → HCl + CH_2_CN process were measured using different experimental techniques and were computed applying transition state theory.^34−36,38−40,42^ Furthermore, photo-detachment of an electron from the Cl^–^···CH_3_CN anion complex was also studied both experimentally and theoretically, thereby probing the neutral system as well.^37,41^ The theoretical studies usually employed the density functional theory, MP2, QCISD(T), and CCSD(T) methods with a triple-zeta basis and investigated only the H abstraction channel.^40−42^ Thus, we have multiple goals in the present study. First, we aim to uncover novel reaction pathways and product channels for the Cl + CH_3_CN reaction and determine their energetics requirements. Second, we plan to move beyond the accuracy of the previous work^40−42^ by using explicitly correlated coupled-cluster theory and basis sets up to quadruple-zeta quality as well as considering effects of correlation beyond the gold-standard CCSD(T) level, core electron correlation, scalar relativity, and spin–orbit coupling. These high-level ab initio results will anchor the energetics of the PES, thereby guiding future analytical PES developments and reaction dynamics simulations. Such dynamics studies have not been reported for the title reaction but are available for the F + CH_3_CN brother system^43−46^ and also for the Cl + CH_4_, C_2_H_6_, and CH_3_NH_2_ cousins.^7−22,25,30^ In these reactions, the HF or HCl rotational distributions are the key dynamics properties, whose accurate determination challenged theory for many years, especially for the HCl product.^14,25,47^ This adds to our motivation to investigate the title reaction to see how the CN ligand affects the HCl rotational distributions. As a first step toward this direction, in the present paper, we give the details of the high-level composite ab initio computations in Section 2, discuss the results on the stationary-point properties along the different reaction pathways in Section 3, and provide the Summary and Conclusions in Section 4.

## 2 Computational Details

The important stationary-point geometries of the potential energy surface of the Cl + CH_3_CN reaction are first determined with the restricted open-shell second-order Møller–Plesset perturbation theory (RMP2)^48^ using the correlation-consistent aug-cc-pVDZ basis set.^49^ The initial structures of the geometry optimizations are based on chemical intuition and previous studies.^23,24,32^ To further optimize the obtained minima and saddle-point structures and compute the harmonic vibrational frequencies, we use the restricted open-shell Hartree–Fock (ROHF)-based unrestricted explicitly correlated coupled-cluster singles, doubles, and perturbative triples (CCSD(T)-F12b)^50^ method with the aug-cc-pVDZ and aug-cc-pVTZ basis sets. We also compute CCSD(T)-F12b/aug-cc-pVQZ single-point energies using the most accurate geometries obtained at the CCSD(T)-F12b/aug-cc-pVTZ level of theory. To achieve subchemical accuracy, we explore further energy contributions as detailed below.

The correction of post-CCSD(T) correlations is obtained as follows:
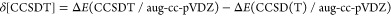
1
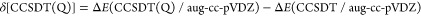
2where unrestricted CCSD(T),^51^ CCSDT,^52^ and CCSDT(Q)^53^ methods are used with the aug-cc-pVDZ basis set and the unrestricted Hartree–Fock (UHF) reference.

The core electron correlation is defined as the difference between the frozen-core (FC) and all-electron (AE) energies:
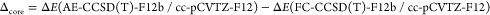
3where the FC approach correlates the valence electrons only, whereas the AE computations also correlate the 1s^2^ (C and N) and 2s^2^2p^6^ (Cl) electrons. The FC and AE energies are obtained at the ROHF-UCCSD(T)-F12b/cc-pCVTZ-F12 level of theory.^50,54^

The scalar relativistic effect is calculated with the following formula:
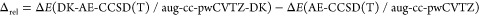
4where second-order Douglas–Kroll (DK)^55^ relativistic energies are computed at the AE-CCSD(T)/aug-cc-pwCVTZ-DK level of theory.^51,56^

Spin–orbit (SO) corrections are determined utilizing the interacting-states approach^57^ using the Davidson-corrected^58^ all-electron multireference configuration interaction^59^ (MRCI+Q) method with the aug-cc-pwCVTZ basis set^60^ and with an active space of 5 electrons in 3 spatial 3p-like orbitals. Higher-order correlation energy effects are estimated by the Davidson correction (+Q). The SO eigenstates are provided by diagonalizing a 6 × 6 SO matrix, where the corrected MRCI energies replace the diagonal elements. The SO corrections are defined as follows:
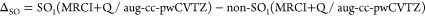
5where SO_1_ and non-SO_1_ are the SO and non-SO ground-state energies, respectively.

All computations are carried out with the Molpro program package,^61^ except the CCSD(T), CCSDT, and CCSDT(Q) computations, where the energies are obtained with MRCC^62,63^ interfaced to Molpro.

The benchmark classical relative energies are calculated as follows:
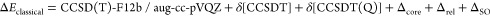
6

The benchmark adiabatic relative energies are computed as follows:
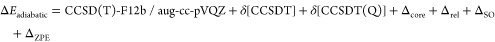
7where Δ_ZPE_ is the zero-point energy correction, which is obtained at the CCSD(T)-F12b/aug-cc-pVTZ level of theory from the harmonic frequency computations.

## 3 Results and Discussion

**Figure 1:**
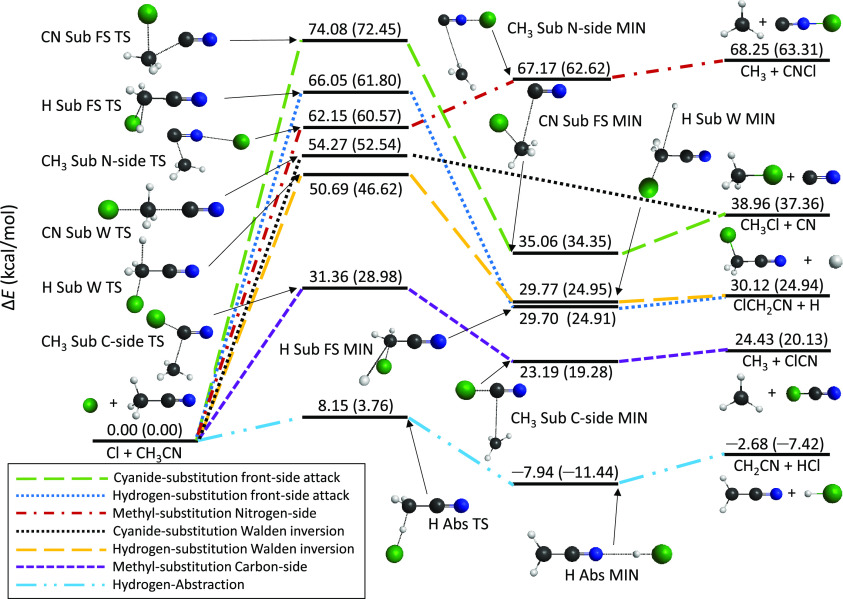
Schematic of the potential energy surface of the Cl(^2^P_3/2_) + CH_3_CN reaction pathways showing the benchmark classical (adiabatic) relative energies of the stationary points.

**Figure 2:**
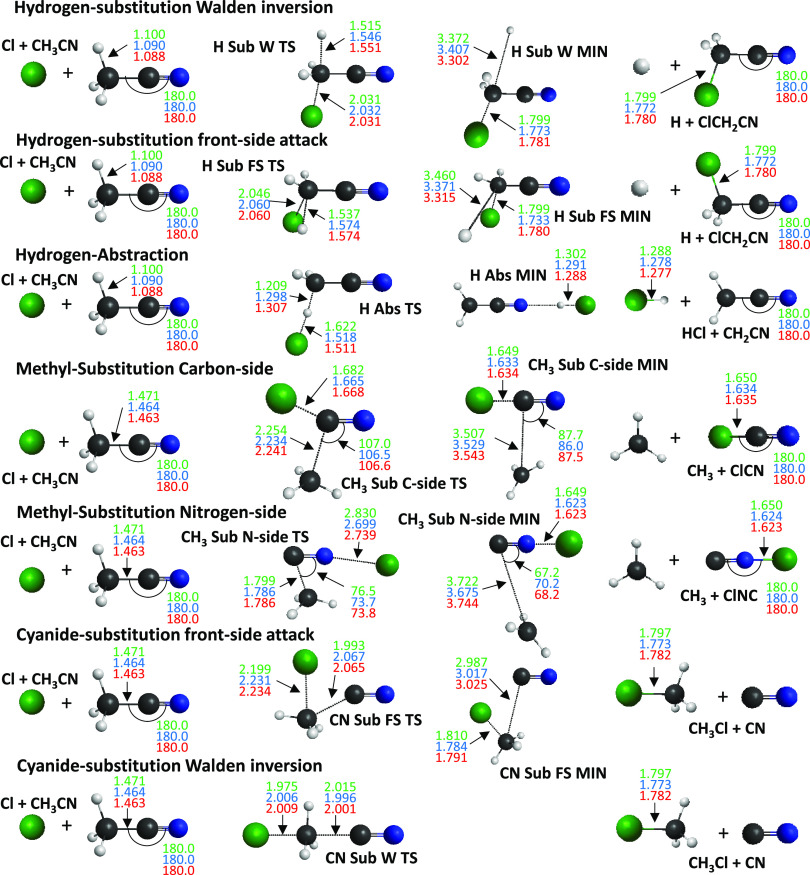
Structures of the stationary points corresponding to the different pathways of the Cl + CH_3_CN reaction showing the most important bond lengths (Å) and angles (degree) obtained with the MP2/aug-cc-pVDZ (green), CCSD(T)-F12b/aug-cc-pVDZ (blue), and CCSD(T)-F12b/aug-cc-pVTZ (red) levels of theory.

The seven pathways of the Cl(^2^P_3/2_) + CH_3_CN reaction are shown in Figure 1 with the benchmark classical and adiabatic energies of the stationary points relative to the reactants. The most important structural parameters of the stationary-point geometries, determined at different levels of theory, are given in Figure 2. The only exothermic reaction channel is the hydrogen abstraction (H Abs) leading to HCl and CH_2_CN. This channel has the benchmark classical (adiabatic) relative energy value of −2.68 (−7.42) kcal mol^–1^. The reaction has a small barrier of 8.15 (3.76) kcal mol^–1^ and a N···HCl bonded complex (H Abs MIN) in the product channel with a *D*_e_ (*D*_0_) dissociation energy of 5.26 (4.02) kcal mol^–1^. All the other reaction pathways are endothermic.

After the hydrogen abstraction, the carbon-side methyl substitution (CH_3_ Sub C-side) has the lowest barrier, 31.36 (28.98) kcal mol^–1^. This channel leads to CH_3_ and ClCN products with Δ*E* (Δ*H*_0_) = 24.43 (20.13) kcal mol^–1^. However, the nitrogen-side methyl substitution (CH_3_ Sub N-side) products (CH_3_ and CNCl) have the highest relative energy, 68.25 (63.31) kcal mol^–1^; thus, this is the most endothermic pathway. The barrier of the N-side reaction is higher by 30.79 (31.59) kcal mol^–1^ than that of the C-side methyl substitution, whereas the *D*_e_ (*D*_0_) values of the CH_3_ Sub complexes are quite similar, 1.24 (0.85) and 1.08 (0.69) kcal mol^–1^.

In the case of the hydrogen substitution (H Sub), leading to H and ClCH_2_CN, the relative energy of the products is 30.12 (24.94) kcal mol^–1^. This reaction can proceed via a Walden inversion (W) transition state (TS) or a front-side attack (FS) TS. The barrier height of the W TS is 50.69 (46.62) kcal mol^–1^, while that of the FS TS is substantially higher, 66.05 (61.80) kcal mol^–1^. The classical relative energy differences between the products and product-like minimum complexes are only 0.35 (W) and 0.42 (FS) kcal mol^–1^; however, considering the ZPE corrections, the *D*_0_ dissociation energies of the complexes are even closer to zero (−0.01 (W) and 0.03 (FS) kcal mol^–1^). Thus, it seems that these shallow product wells do not support stable vibrational ground-state complexes. Similarly to the H Sub, the cyanide substitution (CN Sub) via the Walden TS has a lower barrier than the FS pathway. Moreover, the latter one has the highest barrier of 74.08 (72.45) kcal mol^–1^ among the mechanisms studied in this work. This reaction leads to the CN and CH_3_Cl products with Δ*E* (Δ*H*_0_) = 38.96 (37.36) kcal mol^–1^, and the *D*_e_ (*D*_0_) value of the ClCH_3_···CN complex along the FS pathway is 3.90 (3.01) kcal mol^–1^. Note that we have not found the W MIN for CN Sub.

The diversion of the above-mentioned product–complex dissociation energies is caused by the difference in the chemical interactions between the fragments. For example, the H Abs complex (H_2_CCN···HCl) contains a dipole–dipole interaction that is stronger than the dipole–induced dipole interaction in the CH_3_ Sub complexes (ClCN/CNCl···CH_3_); thus, the *D*_e_ (*D*_0_) value of the H Abs complex is higher. In the case of the H Sub reaction, because of the extremely low polarizability of the hydrogen atom, the ClH_2_CCN···H complexes are quite unstable and their dissociation energies are also low. Both the H Abs (H_2_CCN···HCl) and CN Sub (ClCH_3_···CN) complexes have dipole–dipole interactions, but the dipole moment of the HCl molecule is larger than that of the CN radical; therefore, the H Abs complex is more stable.

The most important structural parameters of the stationary points of the Cl + CH_3_CN reaction are shown in Figure 2. They are determined at different levels of theory: MP2/aug-cc-pVDZ, CCSD(T)-F12b/aug-cc-pVDZ, and CCSD(T)-F12b/aug-cc-pVTZ. The distances obtained with MP2 and CCSD(T)-F12b mainly differ by about 0.01 Å, while the difference between the CCSD(T)-F12b bond lengths determined with the aug-cc-pVDZ and aug-cc-pVTZ basis sets is usually just about 0.001 Å. Greater, even by orders of magnitude, differences can appear in the case of large intermolecular distances because these distance deviations do not cause a significant change in the relative energy values.

**Figure 3:**
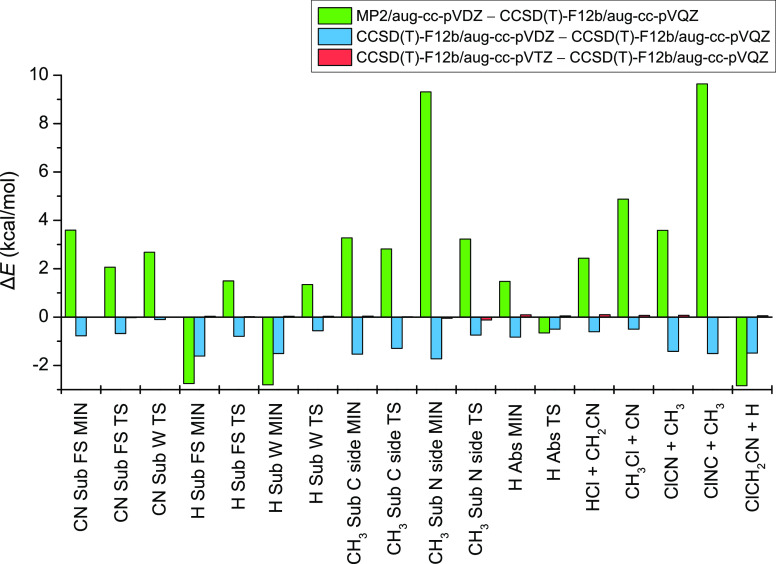
Convergence of the relative energies for stationary points and products of the Cl + CH_3_CN reaction, obtained with different levels of theory: the MP2 method with the aug-cc-pVDZ basis set and the CCSD(T)-F12b method with the aug-cc-pVDZ, aug-cc-pVTZ, and aug-cc-pVQZ basis sets.

The convergence of the relative energies is shown in Table 1 and Figure 3. The average deviation of the MP2/aug-cc-pVDZ and the corresponding CCSD(T)-F12b relative energies is about 3–4 kcal mol^–1^, but in some cases, it can also be even higher than 9 kcal mol^–1^. Consequently, the use of the CCSD(T)-F12b method is needed to reach chemical or subchemical accuracy. In the case of CCSD(T)-F12b, the average absolute difference for the aug-cc-pVDZ relative energies with respect to the aug-cc-pVQZ values is about 1 kcal mol^–1^, whereas the average deviation for the aug-cc-pVTZ relative energies is just about 0.04 kcal mol^–1^, showing the outstanding basis convergence of the explicitly correlated CCSD(T)-F12b method.

**Table 1:** Energies (kcal/mol) at Different Levels of Theory and Their Auxiliary Corrections (kcal/mol) for the Stationary Points and Product Channels of the Cl(^2^P_3/2_) + CH_3_CN Reaction Relative to the Reactants

	MP2	CCSD(T)-F12b										
stationary points	aVDZ^a^	aVDZ^b^	aVTZ^c^	aVQZ^d^	δ[T]^e^	δ[(Q)]^f^	Δ_core_^g^	Δ_rel_^h^	Δ_SO_^i^	classical^j^	Δ_ZPE_^k^	adiabatic^l^
H Abs TS	7.28	7.43	7.98	7.93	–0.36	–0.26	–0.04	+0.07	+0.80	8.15	–4.39	3.76
CH_3_ Sub C-side TS	33.90	29.78	31.09	31.08	–0.64	–0.36	+0.28	+0.18	+0.83	31.36	–2.38	28.98
H Sub W TS	51.62	49.71	50.31	50.27	–0.23	–0.43	+0.21	+0.05	+0.81	50.69	–4.07	46.62
CN Sub W TS	57.51	54.73	54.82	54.83	–1.32	–0.67	+0.65	+0.01	+0.78	54.27	–1.73	52.54
CH_3_ Sub N-side TS	64.25	60.28	60.90	61.02	–0.13	–0.09	+0.77	–0.11	+0.68	62.15	–1.58	60.57
H Sub FS TS	67.41	65.12	65.93	65.92	–0.39	–0.48	+0.18	+0.00	+0.83	66.05	–4.25	61.80
CN Sub FS TS	77.14	74.40	75.05	75.07	–1.49	–0.86	+0.64	–0.10	+0.81	74.08	–1.63	72.45
H Abs MIN	–6.95	–9.26	–8.33	–8.43	–0.45	–0.09	–0.07	+0.27	+0.83	–7.94	–3.50	–11.44
CH_3_ Sub C-side MIN	25.53	20.72	22.29	22.25	+0.02	–0.23	+0.12	+0.20	+0.83	23.19	–3.90	19.28
H Sub W MIN	26.00	27.29	28.83	28.80	+0.15	–0.23	+0.04	+0.18	+0.83	29.77	–4.83	24.95
CH_3_ Sub N-side MIN	75.18	64.14	65.81	65.87	–0.15	–0.05	+0.51	+0.16	+0.83	67.17	–4.55	62.62
H Sub FS MIN	25.98	27.11	28.76	28.73	+0.15	–0.23	+0.04	+0.18	+0.83	29.70	–4.79	24.91
CN Sub FS MIN	38.82	34.44	35.21	35.22	–1.17	–0.45	+0.52	+0.11	+0.83	35.06	–0.71	34.35
HCl + CH_2_CN	–0.78	–3.82	–3.11	–3.21	–0.48	–0.06	+0.00	+0.23	+0.83	–2.68	–4.74	–7.42
ClCN + CH_3_	27.06	22.05	23.55	23.48	+0.02	–0.22	+0.13	+0.20	+0.83	24.43	–4.30	20.13
ClCH_2_CN + H	26.30	27.65	29.19	29.14	+0.15	–0.22	+0.04	+0.18	+0.83	30.12	–5.18	24.94
ClNC + CH_3_	76.55	65.41	66.89	66.91	–0.16	–0.03	+0.52	+0.17	+0.83	68.25	–4.94	63.31
CH_3_Cl + CN	44.32	38.94	39.51	39.44	–1.59	–0.48	+0.60	+0.15	+0.83	38.96	–1.60	37.36

a MP2/aug-cc-pVDZ relative energies obtained at MP2/aug-cc-pVDZ geometries.

b CCSD(T)-F12b/aug-cc-pVDZ relative energies obtained at CCSD(T)-F12b/aug-cc-pVDZ geometries.

c CCSD(T)-F12b/aug-cc-pVTZ relative energies obtained at CCSD(T)-F12b/aug-cc-pVTZ geometries.

d CCSD(T)-F12b/aug-cc-pVQZ relative energies obtained at CCSD(T)-F12b/aug-cc-pVTZ geometries.

e CCSDT – CCSD(T) obtained at CCSD(T)-F12b/aug-cc-pVTZ geometries with the aug-cc-pVDZ basis set.

f CCSDT(Q) – CCSDT obtained at CCSD(T)-F12b/aug-cc-pVTZ geometries with the aug-cc-pVDZ basis set.

g Core correlation corrections obtained as the differences between all-electron and frozen-core CCSD(T)-F12b/cc-pCVTZ-F12 relative energies at CCSD(T)-F12b/aug-cc-pVTZ geometries.

h Scalar relativistic effects obtained as the difference between DK-AE-CCSD(T)/aug-cc-pwCVTZ-DK and AE-CCSD(T)/aug-cc-pwCVTZ relative energies at CCSD(T)-F12b/aug-cc-pVTZ geometries.

i Spin–orbit (SO) corrections obtained as the differences between the SO and non-SO ground-state MRCI+Q/aug-cc-pwCVTZ relative energies at CCSD(T)-F12b/aug-cc-pVTZ geometries.

j Benchmark classical relative energies obtained as CCSD(T)-F12b/aug-cc-pVQZ relative energies + δ[T] (e) + δ[(Q)] (f) + Δ_core_ (g) + Δ_rel_ (h) + Δ_SO_ (i).

k Zero-point energy (ZPE) corrections obtained at CCSD(T)-F12b/aug-cc-pVTZ.

l Benchmark vibrationally adiabatic relative energies obtained as classical relative energies (j) + Δ_ZPE_ (k).

**Figure 4:**
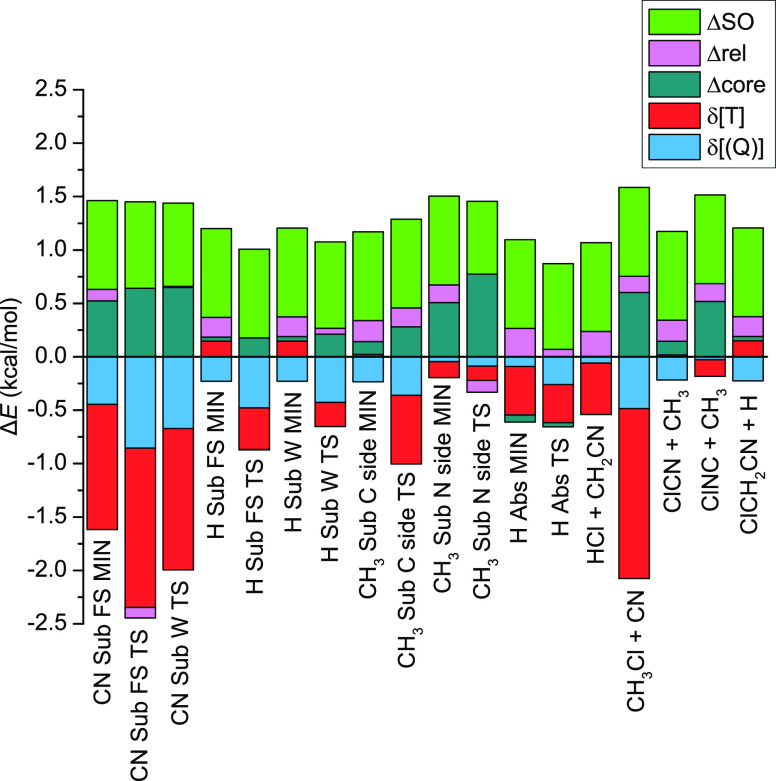
Energy contributions of the post-CCSD(T) (eqs 1 and 2) and core (eq 3) correlations, scalar relativistic effects (eq 4), and spin–orbit corrections (eq 5) for stationary points and products of the Cl + CH_3_CN reaction.

**Figure 5:**
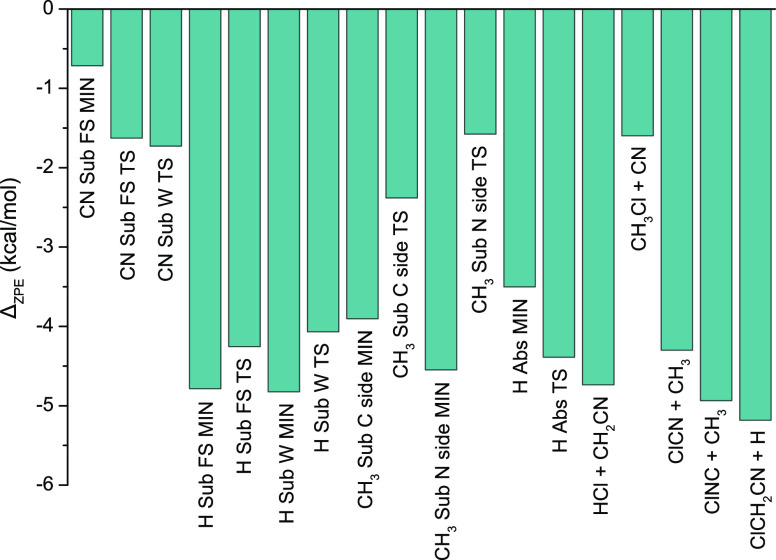
Zero-point energy corrections for the stationary points and products of the Cl + CH_3_CN reaction.

Despite the fact that the large-basis CCSD(T)-F12b computations provide high accuracy, to approach the “exact” energies, some additional energy contributions need to be considered. These auxiliary corrections are shown in Table 1 and Figures 4 and 5. The post-CCSD(T) correlation corrections are usually found to be negative contributions. The δ[CCSDT] correction is typically larger than the δ[CCSDT(Q)] correction, and in most cases, the absolute contribution is around 0–0.5 kcal mol^–1^. However, for the CN Sub, the δ[CCSDT] corrections are above 1 kcal mol^–1^. The core correlation corrections (Δ_core_) are usually small and positive contributions in the range from −0.06 to 0.77 kcal mol^–1^. The scalar relativistic effects (Δ_rel_) are around 0.1 kcal mol^–1^, and generally, these contributions are smaller than the core correlation corrections. The absolute Δ_core_ contributions are always larger for saddle points than minima, except for H Abs, whereas in the case of the Δ_rel_ corrections, the opposite is true without exception. The contribution of the relativistic spin–orbit interaction in the Cl atom is also needed to be taken into account. The energy of the Cl atom decreases with about 0.8 kcal mol^–1^ due to spin–orbit coupling; thus, the relative energy of the stationary points increases with this value. Some differences can appear in the case of the saddle points; for example, for the nitrogen-side CH_3_ Sub TS, the contribution is just 0.68 kcal mol^–1^. This correction will be discussed in more detail later in this section.

Although some corrections have a value greater than 1 kcal mol^–1^, they often balance each other due to the opposite sign. Nevertheless, they are not negligible since in the case of nitrogen-side CH_3_ Sub, for example, the amount of corrections is 1.12–1.33 kcal mol^–1^, so it does not reach chemical accuracy. The harmonic ZPE correction is needed to be considered to get experimentally observable quantities. This correction has the largest effect on the relative energy values, compared to the contributions of the other auxiliary corrections. The absolute ZPE contribution is in the range of 0.7–5.2 kcal mol^–1^ and has an average of 3.5 kcal mol^–1^. The ZPE corrections turn out to be negative in all cases, and the products usually have larger corrections than the corresponding saddle points and exit-channel minima (see Table 1 and Figure 5).

**Figure 6:**
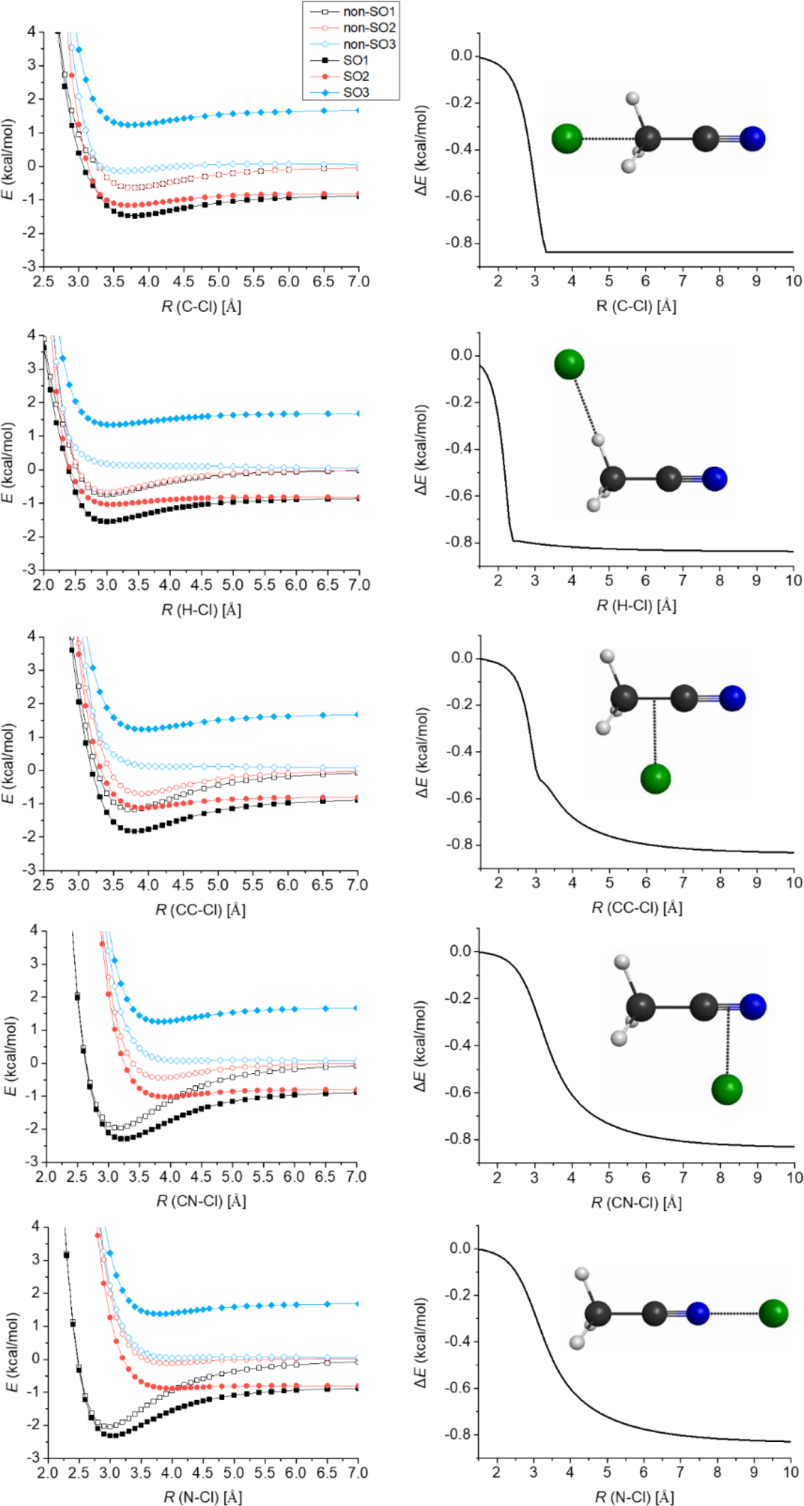
Potential energy curves of the CH_3_CN···Cl system obtained at the MRCI+Q/aug-cc-pwCVTZ level of theory considering five different separation directions: The Cl atom is approaching CH_3_CN from the methyl group along the C_3_ axis (first row), approaching one H atom of the methyl group (second row), approaching perpendicularly the C–C bond (third row) and the C–N bond (fourth row), and approaching the N atom along the C_3_ axis (fifth row). The CH_3_CN unit is kept frozen at its equilibrium geometry. The distance dependence of the difference between the spin–orbit (SO) and non-SO ground-state energies is shown on the right panels.

Because of the above-mentioned spin–orbit (SO) interaction, the non-relativistic ground state of the Cl atom (^2^P) splits into two energy levels, ^2^P_1/2_ and ^2^P_3/2_. The ground ^2^P_3/2_ state has a lower energy by ε/3 than ^2^P, and the excited ^2^P_1/2_ state is higher in energy by 2ε/3, where ε is the SO splitting. As the Cl atom approaches the molecule, the spherical symmetry changes; thus, the fourfold degenerate ground ^2^P_3/2_ state splits into SO ground (SO_1_) and excited (SO_2_) states, and the sixfold degenerate non-relativistic ^2^P state also splits into a non-SO ground (non-SO_1_) and two non-SO excited (non-SO_2_ and non-SO_3_) states. All the resulting states are twofold degenerate. Only the SO_1_ and non-SO_1_ ground states are reactive, i.e., correlate adiabatically with electronic ground-state products. As shown in Figure 6, we consider five different directions of the Cl atom approaching the CH_3_CN molecule. The depth of the van der Waals wells depends on the direction. The shallowest well with a depth of 0.7/0.6 kcal mol^–1^ with/without the SO interaction belongs to the case when the Cl atom approaches the methyl group, while the deepest one with a depth of 1.8/2.4 kcal mol^–1^ is when the Cl atom attacks the molecule from the N atom. As seen, SO coupling plays an important role in the entrance channel and decreases the well depths if the interaction energy is substantial. Furthermore, as also shown in Figure 6, the gap between the SO_1_ and non-SO_1_ potentials decreases as the Cl atom approaches the CH_3_CN molecule, and at small intermolecular distances, the two potentials merge; as a consequence, the SO_1_ – non-SO_1_ energy differences tend to zero.

We have used the Active Thermochemical Tables (ATcT)^64,65^ to compare the computed benchmark vibrationally adiabatic relative energies with the available “experimental” data. Three reaction enthalpies can be compared: The ATcT (present computed) 0 K reaction enthalpies are −7.01 ± 0.17 (−7.42), 20.10 ± 0.13 (20.13), and 38.65 ± 0.09 (37.36) kcal mol^–1^ for H Abs, CH_3_ Sub, and CN Sub, respectively. The CH_3_ Sub reaction gives the best agreement, and the difference between the experimental and the present computed values is only 0.03 kcal mol^–1^. For the H Abs, this deviation is 0.41 kcal mol^–1^, which is still well below chemical accuracy. In the case of CN Sub, the absolute deviation is much greater; it is 1.29 kcal mol^–1^, indicating some issue with both/either theory and/or ATcT.

## 4 Summary and Conclusions

In this study, the different pathways of the Cl + CH_3_CN reaction have been investigated. In addition to the already studied H abstraction, we have also examined the H substitution, CH_3_ substitution, and CN substitution reaction paths. The first mentioned H Abs, leading to the HCl and CH_2_CN products, is the only exothermic (Δ*H*_0_ = −7.42 kcal mol^–1^) reaction; however, it also has a small classical (adiabatic) barrier of 8.15 (3.76) kcal mol^–1^. The H substitution (with the products of H and ClCH_2_CN) can proceed via a Walden-inversion transition state and also a front-side attack TS. The Walden-inversion pathway has a lower barrier by about 15 kcal mol^–1^. This case is similar to the CN substitution, leading to CN and CH_3_Cl products (Δ*H*_0_ = 37.36 kcal mol^–1^): The Walden-inversion barrier height is 54.27 (52.54) kcal mol^–1^, whereas the front-side attack TS has a higher relative energy of 74.08 (72.45) kcal mol^–1^. Two reaction channels are possible in the case of the CH_3_ substitution: The Cl atom can bind to the molecule at the C atom or at the N atom of the CN group, so the products can be CH_3_ + ClCN (Δ*H*_0_ = 20.13 kcal mol^–^^1^) or CH_3_ + CNCl (Δ*H*_0_ = 63.31 kcal mol^–1^). The stationary-point properties have been investigated using a high-level composite ab initio method. We have determined the benchmark classical and vibrationally adiabatic energies using the most accurate CCSD(T)-F12b/aug-cc-pVQZ values refined with the contributions of the post-(T) and core correlation, scalar relativistic effects, spin–orbit coupling, and ZPE corrections. The post-(T) correlation effects are substantial, especially for the CN Sub reactions. The core and scalar relativistic corrections are usually small and positive contributions, while the SO coupling effect almost always means a relative energy increase of 0.8 kcal mol^–1^. Based on this study, in the future, it is possible to develop a global analytical potential energy surface for the Cl + CH_3_CN system, allowing dynamic investigations to gain deeper insight into the atomic-level mechanisms of this multichannel reaction.
